# What we choose to value in the artificial intelligence era of healthcare

**DOI:** 10.1017/S0266462326104061

**Published:** 2026-07-29

**Authors:** Jennifer C. Goldsack

**Affiliations:** Digital Medicine Society (DiMe), USA

Health technology assessment (HTA) exists to help healthcare systems determine what is worth paying for. For decades, HTA has served as a critical bridge between innovation and access. By evaluating the clinical effectiveness, safety, and value of health technologies, HTA agencies help determine which interventions should be adopted, reimbursed, and scaled. By evaluating the clinical effectiveness, safety, and value of health technologies, HTA agencies help determine which interventions should be adopted, reimbursed, and scaled. In doing so, they influence how healthcare resources are allocated and which innovations are developed.

At the 2026 Health Technology Assessment International (HTAi) Annual Meeting (1) in Istanbul, discussions repeatedly returned to the pressures facing HTA. Patients want faster access to innovation. Manufacturers seek recognition of increasingly sophisticated technologies. Governments face growing fiscal constraints. HTA agencies are expected to move more quickly, assess more complex technologies, and make decisions with less certainty than ever before. The result is a growing tension between innovation, access, and affordability that is challenging HTA systems around the world.

Those pressures are evident across every category of health technology, from gene therapies and precision medicines to digital health technologies and artificial intelligence. Consider the therapies reaching healthcare systems today. Cell and gene therapies, precision oncology products, and treatments for rare diseases are often supported by limited evidence, small patient populations, and claims of benefits that may extend decades into the future. HTA frameworks originally developed for chronic therapies with mature evidence packages are increasingly being asked to assess the value of interventions that may cost millions of dollars per patient, even with only a few years of follow-up data.

At the same time, digital health technologies and artificial intelligence are challenging many of the assumptions on which traditional assessment processes were built. Software evolves after launch. Algorithms can drift over time. Clinical performance often depends as much on implementation and workflow integration as on technical accuracy. An AI model updated every few months does not fit neatly within assessment processes that may take years to complete.

The discussions in Istanbul repeatedly highlighted that HTA systems are increasingly being asked to make decisions using evidence frameworks developed for a different era of healthcare. Randomized controlled trials remain the gold standard for establishing causal inference, and they will continue to play an essential role in healthcare decision-making. However, they were never designed to be the sole source of evidence for an ecosystem characterized by rapidly evolving technologies, continuous software updates, increasingly personalized interventions, and unprecedented access to real-world data.

The limitations are becoming increasingly apparent. Traditional trials are expensive, time-consuming, and often exclude large portions of the populations who will ultimately use the technologies being evaluated. Healthcare systems now generate vast quantities of digital data through electronic health records, connected devices, remote monitoring technologies, and routine care delivery. At the same time, advances in artificial intelligence are expanding our ability to generate insights from these data at a scale that would have been impossible only a few years ago.

Regulators are already responding to this reality. Around the world, agencies are embracing novel trial designs, synthetic data approaches, and regulatory sandbox environments to accelerate learning while maintaining appropriate safeguards. Breakthrough and accelerated pathways increasingly recognize the need to balance timely access with continued evidence generation.

In the United States, recent policy developments offer a glimpse of what a more modern evidence ecosystem could look like.

In late 2025, the US Food and Drug Administration launched the Technology-Enabled Meaningful Patient Outcomes (TEMPO) pilot (2), a first-of-its-kind effort to evaluate how digital health technologies perform in real-world settings while generating evidence to support broader adoption. The pilot reflects growing recognition that real-world performance data can complement traditional premarket evidence and help accelerate patient access to promising technologies.

More recently, the Centers for Medicare and Medicaid Services (CMS) and FDA announced the Regulatory Alignment for Predictable and Immediate Device (RAPID) pathway (3), which aligns regulatory and coverage decision-making earlier in the development process for breakthrough medical devices. By bringing regulators and payers together before products reach the market, RAPID seeks to align evidence generation with the needs of regulatory review, coverage, and reimbursement decisions.

Both initiatives reflect a growing recognition that evidence generation, regulatory review, coverage, reimbursement, and implementation are deeply interconnected. When those activities are aligned, healthcare systems can simultaneously generate the evidence needed for regulatory decision-making, reimbursement, and adoption. When they are not, patients wait longer for access, and innovators face uncertainty about what evidence is required to bring new technologies into care.

The implications are particularly important for AI-enabled medical devices. More than 1,500 AI-enabled medical devices (4) have now received authorization from the FDA, yet relatively few have achieved widespread deployment in routine care. The challenge is rarely limited to whether the technology works. Health systems, clinicians, and payers also need evidence that supports implementation, integration into clinical workflows, operational impact, and sustainable reimbursement models.

HTA is uniquely positioned to help answer those questions. Doing so will require agencies to embrace living methodologies, make greater use of real-world evidence, and strengthen collaboration with regulators, clinicians, patients, and policy makers. It will also require investment in workforce development. Many HTA organizations face growing shortages of expertise in data science, artificial intelligence, genomics, and advanced analytics, even as the technologies they assess become increasingly complex.

The discussions in Istanbul left little doubt that the HTA community understands the challenge and is ready to lead. Success, however, will require action far beyond HTA agencies themselves. Policy makers must invest in the data infrastructure needed to support the generation and use of real-world evidence. Regulators, payers, manufacturers, and academic researchers must work together to ensure evidence generation strategies are aligned across the product lifecycle. Patients and clinicians must have a greater role in defining the outcomes that matter most. Modernizing HTA is ultimately a shared responsibility across the healthcare ecosystem.

HTA has always been about determining what society values. In the AI era, that responsibility extends beyond evaluating individual technologies. HTA will help shape the evidence ecosystem itself. The methods it embraces, the outcomes it prioritizes, and the signals it sends to innovators will influence the future of healthcare every bit as much as the technologies it evaluates.

What we choose to value will shape the future of care. Ensuring those decisions remain rigorous, relevant, and responsive to the realities of modern healthcare will require leadership from the entire healthcare ecosystem.

## References

[1] Health Technology Assessment International (HTAi). HTAi 2026 Annual Meeting in Istanbul, Türkiye [Internet]. Edmonton (AB): HTAi; 2026 [cited 2026 Jun 16]. Available from: https://htai.eventsair.com/htai-2026-istanbul-annual-meeting/

[2] U.S. Food and Drug Administration. FDA launches TEMPO: A first-of-its-kind digital health pilot to expand access to chronic disease technologies [Internet]. Silver Spring (MD): U.S. Food and Drug Administration; 2025 Dec 5 [cited 2026 Jun 16]. Available from: https://www.fda.gov/news-events/press-announcements/fda-launches-tempo-first-its-kind-digital-health-pilot-expand-access-chronic-disease-technologies

[3] Centers for Medicare & Medicaid Services. CMS and FDA announce RAPID coverage pathway to accelerate patient access to life-changing medical devices [Internet]. Baltimore (MD): Centers for Medicare & Medicaid Services; 2026 Apr 23 [cited 2026 Jun 16]. Available from: https://www.cms.gov/newsroom/press-releases/cms-fda-announce-rapid-coverage-pathway-accelerate-patient-access-life-changing-medical-devices

[4] U.S. Food and Drug Administration. Artificial intelligence-enabled medical devices [Internet]. Silver Spring (MD): U.S. Food and Drug Administration; [cited 2026 Jun 16]. Available from: https://www.fda.gov/medical-devices/software-medical-device-samd/artificial-intelligence-enabled-medical-devices

